# National survey of patient specific IMRT quality assurance in China

**DOI:** 10.1186/s13014-019-1273-5

**Published:** 2019-04-25

**Authors:** Yuxi Pan, Ruijie Yang, Shuming Zhang, Jiaqi Li, Jianrong Dai, Junjie Wang, Jing Cai

**Affiliations:** 1 Department of Radiation Oncology, Peking University Third Hospital, 49th North Garden Road, Haidian District, Beijing, 100191 People’s Republic of China; 2 Department of Radiation Oncology, Chinese Academy of Medical Science Cancer Institute, 17 Panjiayuan Nanli, Beijing, People’s Republic of China; 3 Department of Health Technology and Informatics, The Hongkong Polytechnic University, Hongkong, People’s Republic of China

**Keywords:** IMRT, Patient-specific QA, National survey

## Abstract

**Background:**

To analyze and present the China’s national survey on patient-specific IMRT quality assurance (QA).

**Methods:**

A national survey was conducted in all radiotherapy centers in China to collect comprehensive information on status of IMRT QA practice, including machine, technique, equipment, issues and suggestions.

**Results:**

Four hundred and three centers responded to this survey, accounting for 56.92% of all the centers implementing IMRT in China. The total number of medical physicists and the total number of patients treated with IMRT annually in these centers was 1599 and 305,000 respectively. All centers implemented measurement-based verification. Point dose verification and 2D dose verification was implemented in 331 and 399 centers, respectively. Three hundred forty-eight centers had 2D arrays, and 52 centers had detector devices designed to measure VMAT beams. EPID and film were used in 78 and 70 centers, respectively. Seventeen and 20 centers used log file and 3D DVH analysis, respectively. One hundred sixty-eight centers performed measurement-based verification not for each patient based on different selection criteria. The techniques and methods varied significantly in both point dose and dose distribution verification, from evaluation metrics, criteria, tolerance limit, and steps to check failed IMRT QA plans. Major issues identified in this survey were the limited resources of physicists, QA devices, and linacs.

**Conclusions:**

IMRT QA was implemented in all the surveyed centers. The practice of IMRT QA varied significantly between centers. An increase in personnel, QA devices and linacs is highly desired. National standard, guideline, regulation and training programs are urgently needed in China for consistent and effective implementation of IMRT QA.

## Background

Patient-specific intensity-modulated radiotherapy (IMRT) quality assurance (QA) is critically important to the successful implementation of IMRT. It ensures correct machine-sided delivery of the prescribed dose by checking the accuracy of dose calculation, plan transfer, and treatment delivery. It is strongly recommended as part of the IMRT clinical process by the professional societies such as the American Association of Physicists in Medicine (AAPM), the American Society for Radiation Oncology (ASTRO), the American College of Radiology (ACR), and the Netherlands Commission on Radiation Dosimetry [1–5]. Guidelines and recommendations for IMRT and VMAT QA have also been published, clearly stating that patient-specific IMRT QA is necessary to ensure patient safety. However, none of these publications addresses the issue of how patient-specific IMRT QA should be performed explicitly [6–9]. Recently, AAPM Task Group (TG) 218 reported recommendation on measurement and analysis methods, and tolerance limits for patient-specific IMRT QA [10].

In the past decade, IMRT has been widely adopted in China due to the pressing clinical needs of advanced radiotherapy technologies for treating a booming population of cancer patients nationwide. IMRT has been implemented in more than 700 centers in China, and volumetric-modulated arc therapy (VMAT) has been introduced in more than 110 centers in a much faster pace than that of the fixed gantry IMRT [11]. Despite these advances, to date, there is no national standard or practice guideline on patient-specific IMRT QA in China.

A Work Group on Commissioning and Patient Specific IMRT QA, responsible for drafting the national standard and guideline of IMRT QA, was established under the guidance of the Radiation Oncology Quality Control Committee, the National Quality Control Center of Cancer Theranostics, and the National Health Commission of the People’s Republic of China in June 2017. This Work Group conducted a national survey in September 2017 on the implementation of patient-specific IMRT QA and multi-center validation test of IMRT QA. Information of IMRT uptake, equipment, delivery techniques, QA devices, QA techniques and methods were collected in the survey from radiotherapy centers in China. The purpose of the survey is to collect comprehensive information on and identify key issues of current IMRT QA practice in China for establishing national guidelines and national multi-center validation tests for implementing patient-specific IMRT QA in China. The Work Group will make recommendations based on the survey results on patient-specific IMRT QA methods, tools and devices, time and frequency, comparison approach, evaluation metrics, criteria and tolerance limits, and data interpretation. These recommendations will be implemented through professional guidelines and government policy to improve IMRT QA practice in China to ensure treatment fidelity and patient safety. In this paper, we report the findings of China’s national survey on patient-specific IMRT QA.

## Methods

This survey was conducted in form of questionnaires October 2017 to December 2017. A questionnaire was sent out to the members of the IMRT QA Work Group in each province, autonomous region and municipality directly under the Central Government of China (excluding Hong Kong and Macao Special Administrative Regions and Taiwan province), and then was distributed to radiotherapy centers that have implemented IMRT. Once completed, the questionnaires were collected via email and verified for each item. In case the questionnaire response was found to be substandard, a list of identified problems will be complied and sent back to the respondents via email for clarifications.

In this survey, IMRT was defined as the inversely-planned intensity-modulated radiotherapy techniques. All linac-based IMRT delivery techniques, static and rotating gantry (including TomoTherapy), were included in the survey. Survey questions covered the general information about the radiotherapy centers, medical physicists, IMRT delivery techniques, equipment, patient characteristics, patient-specific IMRT QA details, problems, and suggestions. The patient-specific IMRT QA details included dose verification tools and methods, normalization, dose threshold, data interpretation, tolerance and action limits, method of checking failed IMRT QA plans, Multi-leaf Collimator (MLC) QA, et al.

Descriptive statistics were performed for all variables using SPSS (IBM Corp, Armonk, NY, USA) for Windows, version 22.

## Results

### Responding centers and patients treated

**Fig. 1:**
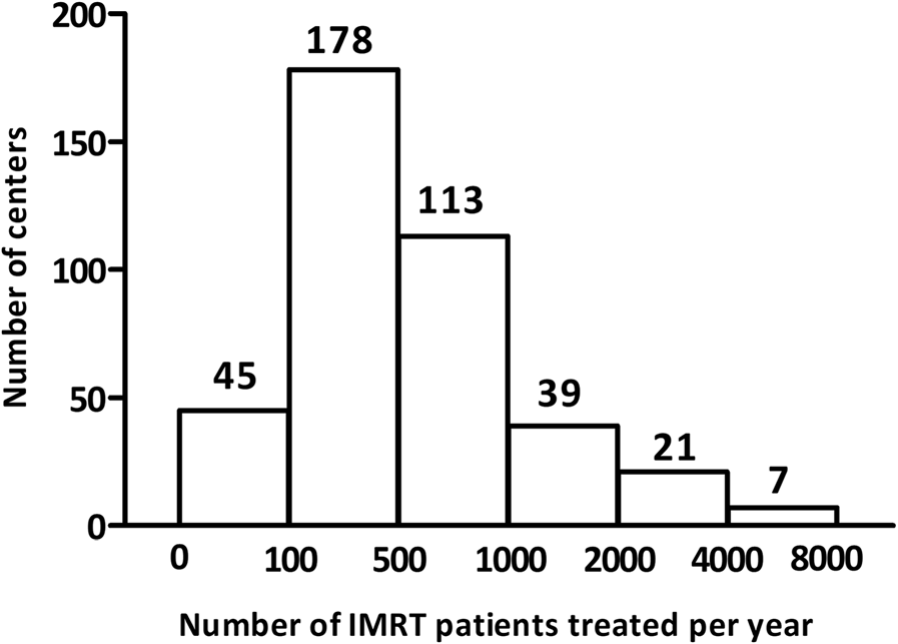
Number of IMRT patients treated per year in the responding centers

Four hundred and three centers responded to this survey, accounting for 56.92% of the centers that are currently practicing IMRT in China. Among the 403 centers, 152 centers have also implemented VMAT. A wide range of IMRT experience was reported, ranging from greater than ten years to less than three months. The 403 responding centers included 41 cancer hospitals and 362 general hospitals; or 100 academic hospitals and 303 non-academic hospitals. Three hundred five thousand patients were treated with IMRT per year in the responding centers (Fig. 1). The most commonly treated sites were lung, breast, cervical, nasopharyngeal, esophageal, and rectal cancer.

### Medical physicists

**Fig. 2:**
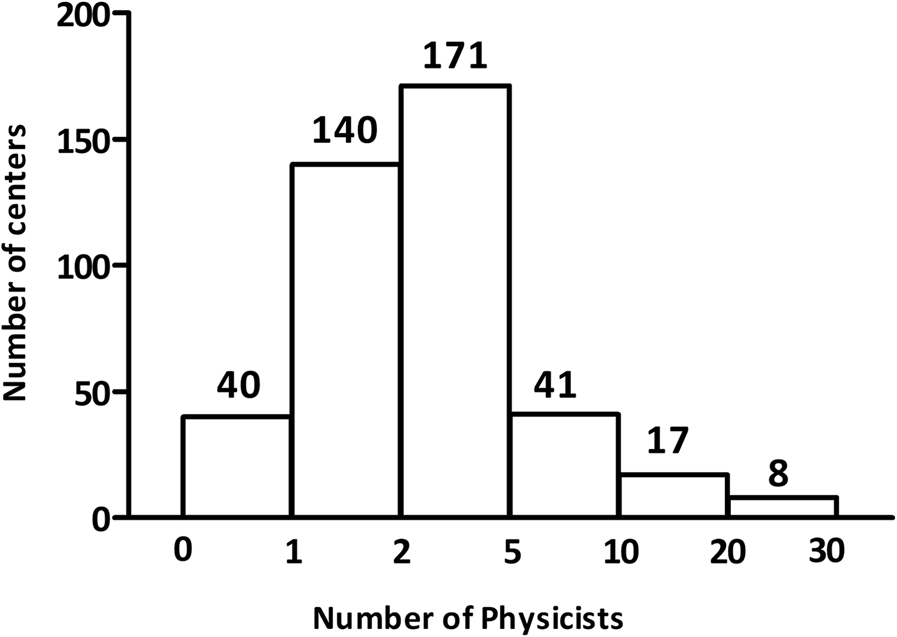
Number of physicists in the responding centers

**Fig. 3:**
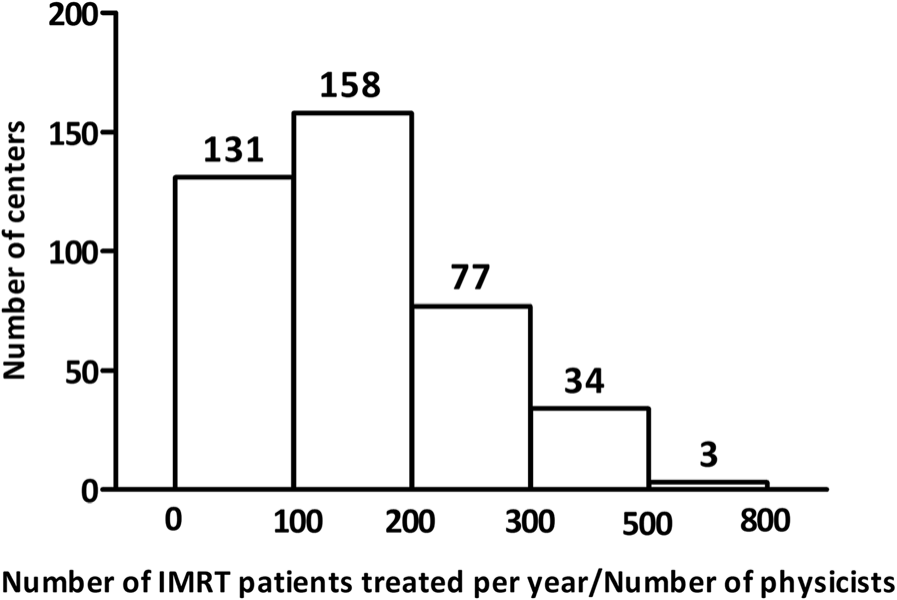
Number of IMRT patients treated per physicist in the responding centers

In all responding centers, medical physicists are reported to be responsible for the IMRT QA program. Radiotherapists and clinical engineers are also involved in IMRT QA under the supervision of medical physicists in 133 centers and 27 centers, respectively. Regardless of the QA performer, checking and approving of the IMRT QA results are performed by medical physicists. There are a total of 1599 medical physicists in the 403 responding centers (Fig. 2). The number of IMRT patients per physicist is shown in Fig. 3. More than 100 IMRT patients were treated per physicist in 272 centers.

### Linacs and TPS

**Fig. 4:**
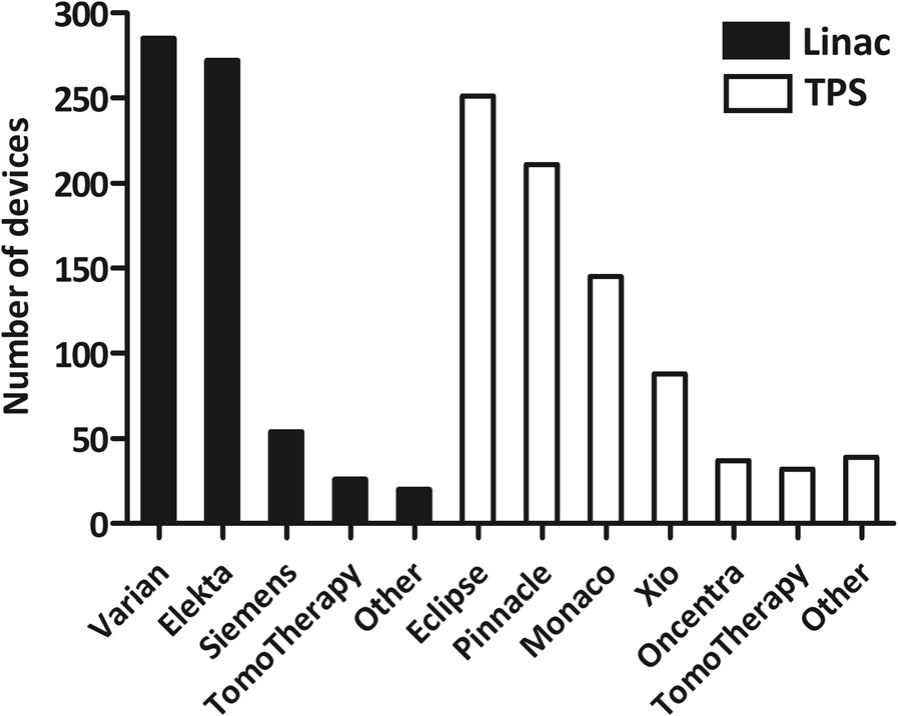
Number of linear accelerators and treatment planning systems in the responding centers

**Fig. 5:**
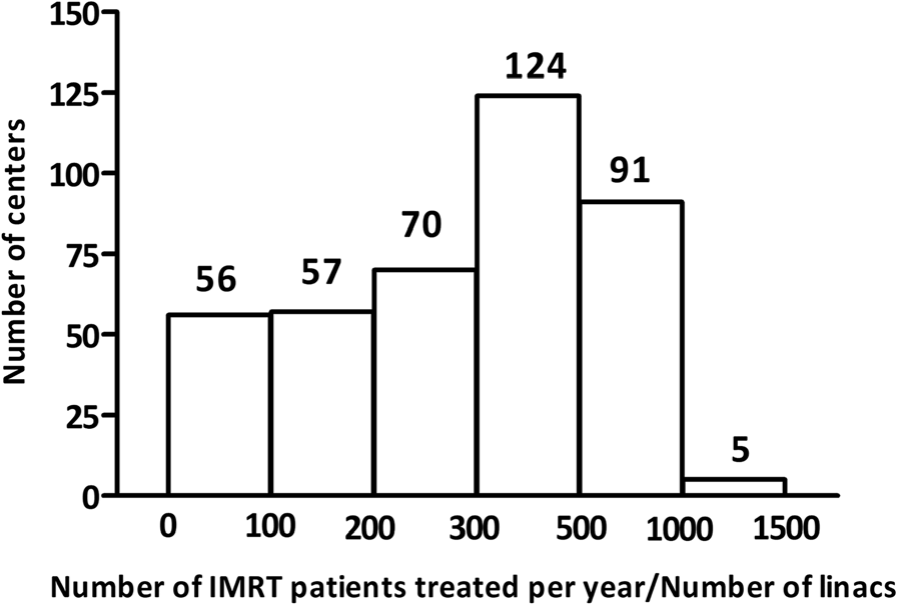
Number of IMRT patients treated per linac in the responding centers

Figure 4 shows the statistics of linear accelerators and treatment planning systems (TPS) used for IMRT. There are a total of 655 linacs and 819 TPS workstations in the responding centers. Some centers used linacs from more than one manufacturer. The number of centers that has 1, 2, 3, 4, and ≥ 5 linacs is 277, 92, 18, 13, and 17, respectively. The number of centers that has 1, 2, 3, 4, and ≥ 5 TPSs is 257, 71, 32, 10 and 33, respectively. The number of IMRT patients treated per linac is shown in Fig. 5. In more than half of the centers, the number of IMRT patients treated per linac was greater than 300.

### Verification techniques and methodologies

Table 1 summarizes the verification techniques and methodologies used for IMRT QA. All responding centers used measurement-based verification method. Three hundred thirty one centers used point dose verification, of those 4 centers used point dose verification only without dose distribution verification. Ion chambers with different sizes (range: 0.01 cc to 0.6 cc) were used for point dose verification. For fixed gantry IMRT QA using 2D array devices, 107 centers used perpendicular field-by-field (PFF) verification to improve efficiency when the IMRT QA results were out-of-tolerance using the perpendicular composite (PC) method. All the responding centers that have implemented VMAT (*n* = 152) applied true composite (TC) method for VMAT QA using Delta4, ArcCheck, Octavious with dedicated phantom, film, Compass or EPID, etc.

**Table 1:** Verification techniques and methodologies for patient specific IMRT QA in China

Items				Number	Percentage (%)
Techniques	Point dose	Tools	Ion chamber	331	82.1
	2D dose	Tools	2D diode or chamber arrays	348	86.4
			EPID	78	19.4
			Film + diode or ionization chamber arrays	58	14.4
			Film	12	3.0
	3D dose	Tools	ArcCheck or Delta 4	52	12.9
Methodologies	Point dose	Location of ion chamber	Isocenter	198	49.1
			Maximum dose point	67	16.6
			Uniform high dose region	39	9.7
			Isocenter or a uniform high dose region	42	10.4
			5 cm for 6 MV, 10 cm for 10 MV plans	29	7.2
		Measurement value	Mean dose to ion chamber volume	171	42.4
			Point dose (effective measurement point)	172	42.7
		Tolerance limits	2%	3	0.7
			3%	356	88.3
			4%	1	0.2
			5%	21	5.2
			Other	19	4.7
			No response	17	4.2
		Action limits	2%	35	8.2
			3%	154	38.2
			5%	78	19.4
			10%	1	0.2
			Other	20	5.0
			No response	161	40
	2D, 3D dose	Delivery methods	Perpendicular field-by-field (PFF)	190	47.1
			Perpendicular composite (PC)	258	64.0
			True composite (TC)	110	27.3
			PFF after PC failure	107	26.6
		Orientation of film/array	Coronal	340	84.4
			Sagittal	35	8.7
			Transverse	41	10.2
		Absolute dose calibration	Before each IMRT QA session	115	28.5
			Weekly	84	20.8
			Monthly	128	31.8
			Every 3 months to one year	70	17.4
			Never	3	0.7
		Grid size	1 mm	39	9.7
			2 mm	155	38.5
			2.5 mm	50	12.4
			3 mm	190	47.1
			4 mm	67	16.6
			5 mm	2	0.5
			Varied with TPS, delivery techniques	75	18.6
		Reference distribution	Measured dose	205	50.9
			Calculated dose	282	70.0
		Dose algorithm	Pencil beam	108	26.8
			Convolution/and superposition	278	69.0
			Monte Carlo	140	34.7
		Evaluation metrics	Dose difference (DD) at multiple points	168	41.7
			Distance-to-agreement (DTA),	155	38.5
			Gamma pass rate	353	87.6
			Profiles or isodose distributions	157	39.0
			Anatomy-based 3D dose distributions and DVHs	20	5.0
		Tolerance limits	90%	38	9.4
			95%	293	72.7
			93%	1	0.2
			No response	85	21.1
		Action limits	90%	35	8.7
			95%	262	65.0
			80%	2	0.5
			No response	118	29.3
		Gamma criteria	2% DD	35	8.7
			3% DD	305	75.7
			4% DD	17	4.2
			5% DD	57	14.1
			1 mm DTA	8	2.0
			2 mm DTA	50	12.4
			3 mm DTA	300	74.7
			4 mm DTA	15	3.7
			5 mm DTA	1	0.2
		Normalization point	Maximum dose point	207	51.4
			Isocenter	166	41.2
			Other points in the high dose plateau region	80	19.9
		Normalization modes	Global normalization	306	75.9
			Local normalization	78	19.4
		Dose analysis modes	Absolute	225	55.8
			Relative	235	58.3
			Both	85	21.1
		Dose thresholds	10%	286	71.0
			20%	33	8.2
			5% or 15%	38	9.4
Reasons and actions		Reasons for failed QA	Plan being too highly modulated	287	71.2
			Dose measurement point in a high gradient region	232	57.6
			Inaccurate phantom set up	195	48.4
			MLC positioning uncertainty	201	49.9
		Actions for failed QA	Checking the verification plan	296	73.4
			Checking the VS, TPS and delivery system	353	87.6
			Re-measure or design verification plan again	317	78.7
			Previous plan verification	151	37.5
			Communicated with physicians	146	36.2
			Re-plan	199	49.4
MLC QA		Frequency	Monthly	242	60.0
			Weekly	131	32.5
			Daily	66	16.4
			Every season, half year or one year	53	13.2
			Never	33	8.2
Audits and clinical trial		Type	External audits or inter-institution comparison	168	41.7
			Clinical trial credential	13	3.2
Issues of IMRT QA		Type	Lack of physicists	174	43.2
			Lack of time	230	57.1
			Lack of QA devices	196	48.6
			Lack of linacs	182	45.2

### Selection criteria of measurement-based QA

235 (58.3%) centers performed measurement-based IMRT QA for all of their IMRT patients, while 168 centers (41.7%) only performed measurement-based IMRT QA for selected patients. The selection criteria varied between centers and included a wide range of factors such as tumor site, plan complexity, prescription dose, fractionation, normal tissue tolerances, treatment delivery technique, intent of treatment, availability of treatment machine, verification tools, physicist time, patient’s economic status, reimbursement, physician and/or patient’s preference, etc. Most of these centers performed patient-specific IMRT QA for all of their IMRT patients during the first year of IMRT implementation (or for the first 100 IMRT patients), after which they randomly selected patients for IMRT QA with a sampling rate ranging from 20 to 60%.

For patients that measurement-based IMRT QA were not performed, 16 centers performed calculation-based verification, but majority of the centers did not perform any other type of verification. All centers performed measurement-based IMRT QA for hypo-fractionation radiotherapy, SBRT and SRS plans.

### Device calibration, reference distribution and dose calculation

The frequency of absolute dose calibration for diode/ion chamber arrays varied between centers. Three centers had never performed absolute dose calibration after commissioning. Measured dose was used as reference in 205 centers, calculated dose was used as reference in 282 centers, and interpolation of measured dose was used as reference in 195 centers. Pencil beam, convolution/and superposition and Monte Carlo based dose calculation algorithms were used in 108, 278 and 140 centers, respectively. Grid size of 1, 2, 2.5, 3, 4 and 5 mm was used for dose calculation in TPS in 39, 155, 50, 190, 67 and 2 centers. 1–4 mm was used in 75 centers based on different treatment planning system, delivery technique, or target size.

### Evaluation metrics, tolerances and action limits

Different evaluation metrics, tolerance and action limits were used for point dose verification and 2D dose verification. Seventy seven centers evaluated the concordance between the calculated and the measured dose distributions with different metrics and criteria in different dose gradient regions.

The gamma criteria implemented for evaluating IMRT QA varied between centers. Dose difference (DD) of 2–5% and dose to agreement (DTA) of 1–5 mm was used in these centers. The most commonly used DD/DTA value for gamma criteria was 3%/3 mm. For normalization methods, 207 centers used maximum dose point as the normalization point, 166 centers used isocenter and 80 centers used other points in the high dose plateau region. Global/local normalization was used in 306 and 78 centers, respectively. Dose analysis mode and threshold were also different between centers.

### Causes and actions for failed IMRT QA results

The most common causes for failed IMRT QA cases were over modulation, point dose measurement in a high dose gradient region, incorrect QA phantom setup, and MLC leaf position uncertainty. Other causes included TPS beam modeling error, QA planning error, small field or narrow long field, QA device error, linac output error, IMRT QA analysis error, and laser issues.

Most centers reported difficulties in analyzing root causes and providing solutions for failed IMRT QA cases. The methods of investigating failed IMRT QA included checking the verification plan, checking the verification system, planning system, and delivery system, repeating measurement, and repeating the entire IMRT QA verification. Three hundred forty nine centers checked the treatment machine consistency, verification system and treatment planning system performance, 195 centers edited the IMRT plan based on the results of IMRT QA, and 144 centers discussed with the radiation oncologist to make a clinical decision.

### MLC QA, external audits, multiple institution comparison, and clinical trials

The frequency of MLC QA varied between centers. Thirty three centers did not perform MLC QA. The MLC tests, including leaf calibration and position accuracy, were performed using film, EPID, 2D array, graph papers or log files. One hundred sixty eight centers reported participation in external IMRT audits or inter-institution comparison, and 13 centers were credentialed for clinical trials with IMRT.

### Issues and suggestions

This survey revealed a significant issue of limited resources in physicist staffing, time, QA device, and treatment machine. In some centers, IMRT QA was voluntarily performed by medical physicists without salary compensation for working overtime. There was no reimbursement for IMRT QA in majority of the centers. One hundred thirty seven centers performed IMRT QA before the first treatment for hypo-fractionated treatments, SRS and SBRT. Two hundred fifty one centers performed IMRT QA during the first three fractions for conventional fractionated treatment. IMRT QA was performed during working hour in day time in 124 centers, in the evening in 177 centers, and on weekend in 283 centers.

The most concerning issues on IMRT QA devices and techniques included the optimal size of ion chamber for point dose measurement, the accuracy and comparability of various techniques and devices with different hardware and software. IMRT QA was largely considered time-consuming, complex and cumbersome in this survey. Easy-to-use devices with high resolution and high efficiency are highly desired. The clinical significance of IMRT QA results was unclear and it is difficult to appreciate for current 2D, phantom-based IMRT QA techniques. There was a lack of 3D anatomy-based IMRT QA devices and techniques.

## Discussion

A national survey on patient-specific IMRT QA has been successfully conducted in China, including 56.92% of the radiotherapy centers that are currently practicing IMRT in China. Overall, the survey results showed that significant variations exist in the implementation of patient-specific IMRT QA among radiotherapy centers, including techniques, equipment, evaluation criteria, etc. This finding reflects the great need of regulations in China for IMRT practice accreditation, certification, audit, examination and monitoring. Technical guidance, support and cooperation, and training programs should be continually implemented to improve the IMRT QA practice nationally, including training programs from QA device and linac manufacturers. The survey results also implies the urgent need to establish medical physicist profession in China.

It is revealed in this survey that burden of IMRT QA is considerably high for medical physicists in China. According to a framework published by American Society for Radiation Oncology (ASTRO) [12], the required number of physicists and dosimetrists for IMRT,IGRT,SRS,TBI and SBRT treatment equals to the patient number multiplied by the coefficient 0.008 and 0.005, respectively. In China, physicist and dosimetrist are not separated and collectively referred to as physicist. Thus, 1.3 physicists(/dosimetrists) are needed for every 100 patients. However, this survey showed that more than 100 IMRT patients were treated per physicist in 272 centers (67%), reflecting the serious workload problem for medical physicist in China. In addition, in more than half of the centers, the number of IMRT patients treated per linac was larger than 300. Considering that there are also many other patients treated in one linac, such as patients treated with 3D-CRT, SBRT etc., implying that the machine is fully occupied for patient treatment during working hours and physicists can only do IMRT QA in the evening or during weekends in most of the centers. To address this significant challenge of limited machine time for IMRT QA, some centers chose the strategy of performing measurement-based IMRT QA for selected patients only after a few years of practicing IMRT.

The survey yielded comprehensive data on current practice of IMRT QA in China, including techniques, equipment, manpower, reimbursement model, etc., providing the evidence foundation for the Work Group on Commissioning and Patient Specific IMRT QA to develop China’s national guidelines for implementing patient-specific IMRT QA. Based on the survey results, the Work Group has developed a series of recommendations for national guidelines for IMRT QA practice in China. Representative recommendations for dose verification and action response to IMRT QA failure are shown below:

### Dose verification

For point dose verification, leakage current should be corrected when small volume ion chambers are used in point dose verification [8, 13]. The ion chamber with adequate spatial resolution should be selected and placed in a plateau dose region, considering of the dose gradient and positioning errors. In general, the dose gradient across the ion chamber for homogeneous dose distributions should be less than 5% of the mean dose to the chamber. For SBRT\SRS, it should be as close as possible to the criteria. The calculated dose to the chamber volume, instead of dose to the effective measurement point or middle of the chamber active volume should be compared with the measured dose.

For planar dose verification, if the angular dependence of 2D array is negligible or can be corrected accurately, the TC measurements can be used. Otherwise, the measurements should be performed using PFF method due to anisotropic dose response of the array detectors [14–16]. The PC method should not be used due to the possibility of masking delivery errors. No significant correlation was observed between PFF 3%/3 mm DTA and the actual 3D dose differences [17–20]. A confidence limit difference of 12.4 and 7% for TC and PFF was noted in the TG-119 report, respectively [3]. So, tools for patient anatomy-based 3D verification and specially designed VMAT QA are highly desirable [21–25].

For gamma index analysis, the evaluated dose distribution should have the same or higher resolution than the reference distribution [26]. For the 282 centers that used calculated dose as the reference distribution, the comparison accuracy was compromised if no interpolation of measured dose distribution was used. When measured dose distribution is used as reference, small calculation grid size should be used. Dose calculation grid size larger than 3 mm is not appropriate for IMRT QA.

Dose difference, DTA, dose profiles and isodose distribution should be reviewed in addition to the gamma pass rate. Furthermore, not only the failure percentage, but also the maximum, average gamma value, and gamma distribution should be reviewed [11]. It is difficult to establish the acceptance limits for IMRT QA because different delivery systems, planning systems, and verification devices are used [27, 28]. Analyzing gamma pass rate with different dose difference/DTA criteria is useful to find the sources and judge the impact of discrepancies. Stricter criteria of 3%/2 mm, even 2%/2 mm should be used, as the experience and confidence increase in IMRT QA. Some centers used locally defined limits varying with cancer site and plan complexity [29]. 10% action limit for point dose verification, 5 mm DTA limits, and 80% action limit for gamma analysis is not acceptable. The European Society for Radiotherapy and Oncology (ESTRO) recommended tolerance and action limits of 3 and 5% for ion chamber measurements [30]. AAPM recommended that tolerance and action limits should be within ≤2% and ≤ 3%, respectively [11]. Planar dose verification using a 2D array with the detector spacing of 7 mm could not detect MLC leaf position errors smaller than 2 mm, with 3%/3 mm criteria and a 90% gamma passing rate [31]. The average deviations in D_95%_ of target, D_0.1cc_ of the spinal cord reached 8 and 12%, respectively, in complex head and neck plans for systematic leaf position errors of 1 mm [32]. Therefore, tighter tolerances should be used together with accelerator and MLC QA [33, 34].

In addition, absolute dose mode should be used for IMRT QA analysis because considerable differences may go undetected using relative dose mode. The absolute dose calibration of the ion chamber or diode arrays should be performed before each measurement, in order to rule out the influence of detector response and accelerator output variation. Global normalization should be used due to its clinical relevance. The normalization point should be placed in a high dose, low gradient region, often the maximum dose point, not necessarily isocenter of the plan, especially when the isocenter is located in the low dose or high gradient region [35].

### Action response to IMRT QA failure

If IMRT QA failed, medical physicist should systematically review the dose difference, DTA, gamma index, isodose distribution, dose profile, structure specific dose distribution and DVH when available, to determine if the dose deviations are clinically acceptable. A comprehensive root causes analysis should be performed to determine the reasons for these discrepancies and find the solution to them. It may be necessary to check the clinical plan, QA plan, QA device, setup, and/or measure with a different measurement device or different geometry. Medical physicists should understand the characteristics and performance of their QA tool, implementation details, and test its accuracy. If the modulation of the failed plan is much more complex than usual, planning with less complex intensity patterns should be considered. If the gamma passing rate is systematically lower than the recommended action limits, then the dose differences should be thoroughly reviewed, using local normalization and tighter criteria, to find subtle regional discrepancies.

The IMRT workflow should be thoroughly investigated, including the TPS beam modeling and commissioning, QA planning, QA device and linac performance testing, and end-to-end tests. Gamma passing rates should be tracked among patients for the same sites, to differentiate if the errors are specific for a treatment site, or delivery equipment. In addition, patient-specific verification QA for previous cases, the multiple center comparison or independent validation tests can also be performed to help identify the sources of errors.

The survey also revealed issues that need to be further investigated and discussed. For example, tools such as EPID and log file that can simplify the set-up, measurement or analysis of IMRT QA should be encouraged [36]. While there are controversies on the value and methods of patient-specific IMRT QA [37–40], especially whether computation can replace measurements, a significant increase is expected in calculation-based verification. For plans that the measurement-based IMRT QA was not performed, calculation-based verification should be considered, together with systematic QAs of linac and TPS [41]. Furthermore, reimbursement of IMRT QA should be addressed and made known to physicians, department directors and hospitals, as well as to the administration and public.

## Conclusions

A national survey on patient-specific IMRT QA was successfully conducted in China. Patient-specific IMRT QA is implemented in all surveyed radiotherapy centers, but the practice varied significantly between centers. The survey shows that IMRT QA is a significant burden to IMRT practice in China, largely due to the limited resources in manpower, equipment and machine time. National standard and guideline, regulation and training programs for IMRT QA are urgently needed in China to ensure effective implementation of IMRT in high quality consistently.

## Abbreviations

AAPM: The American Association of Physicists in Medicine
ACR: The American College of Radiology
ASTRO: The American Society for Radiation Oncology
ESTRO: The European Society for Radiotherapy and Oncology
IMRT: Intensity-modulated radiation therapy
MLC: Multi-leaf Collimator
PC: Perpendicular composite
PFF: Perpendicular field-by-field
QA: Quality assurance
TC: True composite
